# Privacy Attitudes toward Mouse-Tracking Paradata Collection

**DOI:** 10.1093/poq/nfad034

**Published:** 2023-08-09

**Authors:** Felix Henninger, Pascal J Kieslich, Amanda Fernández-Fontelo, Sonja Greven, Frauke Kreuter

**Affiliations:** Graduate Student at the Chair for Statistics and Data Science in Social Sciences and the Humanities, Faculty of Mathematics, Informatics and Statistics, Ludwig-Maximilians-Universität München, Munich, Germany; and Research Affiliate, Mannheim Centre for European Social Research, University of Mannheim, Mannheim, Germany; Research Affiliate, Mannheim Centre for European Social Research, University of Mannheim, Mannheim, Germany; Postdoctoral Researcher, Departament de Matemàtiques, Universitat Autònoma de Barcelona, Barcelona, Spain; and Research Affiliate with Chair of Statistics, School of Business and Economics, Humboldt-Universität zu Berlin, Berlin, Germany; Professor at the Chair of Statistics, School of Business and Economics, Humboldt-Universität zu Berlin, Berlin, Germany; Professor at the Chair for Statistics and Data Science in Social Sciences and the Humanities, Ludwig-Maximilians-Universität München, Munich, Germany; and Professor, Joint Program in Survey Methodology, University of Maryland, College Park, MD, US

## Abstract

Survey participants’ mouse movements provide a rich, unobtrusive source of paradata, offering insight into the response process beyond the observed answers. However, the use of mouse tracking may require participants’ explicit consent for their movements to be recorded and analyzed. Thus, the question arises of how its presence affects the willingness of participants to take part in a survey at all—if prospective respondents are reluctant to complete a survey if additional measures are recorded, collecting paradata may do more harm than good. Previous research has found that other paradata collection modes reduce the willingness to participate, and that this decrease may be influenced by the specific motivation provided to participants for collecting the data. However, the effects of mouse movement collection on survey consent and participation have not been addressed so far. In a vignette experiment, we show that reported willingness to participate in a survey decreased when mouse tracking was part of the overall consent. However, a larger proportion of the sample indicated willingness to both take part and provide mouse-tracking data when these decisions were combined, compared to an independent opt-in to paradata collection, separated from the decision to complete the study. This suggests that survey practitioners may face a trade-off between maximizing their overall participation rate and maximizing the number of participants who also provide mouse-tracking data. Explaining motivations for paradata collection did not have a positive effect and, in some cases, even reduced participants’ reported willingness to take part in the survey.

## Introduction

Collecting cursor movements during a survey provides researchers with a rich and versatile data source that goes beyond the responses to provide information regarding their genesis (Horwitz, Kreuter, and Conrad 2017). This method, known as mouse tracking, holds the potential to identify problematic questions and items, as well as individuals who struggle with them, and could be a foundation for real-time adaptive interventions (Horwitz et al. 2019), adding to online questionnaires the guidance a human interviewer would provide in other survey modalities (De Leeuw 2005; Tourangeau, Conrad, and Couper 2013).

Mouse tracking has received substantial attention in many behavioral and cognitive disciplines over the past decade (see reviews by Freeman 2018; Stillman, Shen, and Ferguson 2018) and is increasingly considered a valuable data source in both survey and user interface research. It is a member of the growing family of paradata methods, which provide additional information concerning the response process beyond the collected answers themselves (Kreuter 2013; McClain et al. 2019). In surveys, these methods help discover issues with the data and the underlying instrument. Stieger and Reips (2010), for example, used excessive mouse movements as a flag for potentially problematic datasets. Horwitz, Kreuter, and Conrad (2017) coded specific cursor movement patterns and demonstrated that these predicted response difficulties. Horwitz et al. (2019) automatically extracted features from cursor trajectories and showed that several characteristics were sensitive to (induced) respondent difficulty. Most recently, Fernández-Fontelo et al. (2023) further improved detection of difficult items using machine learning.

However, because they provide a continuous, moment-by-moment log of a participant’s every interaction with a survey instrument at a high temporal resolution, cursor movements may reveal information respondents did not intend to disclose, such as responses they considered but ultimately changed; by including a component of (involuntary) motor behavior, they enable individual behavioral profiling and recovery of demographic data (Leiva, Arapakis, and Iordanou 2021), respondents’ emotional state (Yamauchi and Bowman 2014), or even health status (Allerhand et al. 2018). These possible applications go substantially beyond other paradata sources, such as time stamps that only reveal the overall response time for an item. Because of its potential to reveal information that goes beyond survey responses, mouse tracking may require participants’ explicit consent that their movements be recorded and analyzed. The need for consent, in turn, may affect the willingness of participants to take part in the survey—if prospective respondents are reluctant to complete the questionnaire if further data are gathered, paradata collection may do more harm than good. Worse still, specific subgroups may decide to drop out at higher rates than others, skewing survey results through selective nonresponse (Plutzer 2019).

There are currently no widely accepted standards for eliciting consent for paradata collection.^1^ The resulting gray area leaves it to individual researchers and institutional ethics review to decide if and how to collect consent, and how to explain the additional data collection: if they communicate their aims well, respondents can decide whether they consider the benefits commensurate to the potential privacy invasion, which could lead to higher consent rates (as in Kunz and Gummer 2019). This line of reasoning is formalized in Contextual Integrity Theory (Nissenbaum 2011, 2004, 2018), which states that privacy violations occur when context-specific informational norms are broken. Embedding a request for data in an appropriate context may thus increase the acceptance of a “data flow.”

Contextual Integrity Theory posits that norms around data usage are translated from everyday transactions and depend on the goals and ultimate use of information. Applied to online surveys, respondents may readily accept that researchers analyze their answers, as they would if the questionnaire was printed, but paradata collection might appear as if the interviewer were watching over their shoulders while they made their choices, and might entail a corresponding reluctance. On the other hand, interviewers are frequently in a position to observe paradata, ranging from verbal pauses to puzzled expressions, and arguably few participants go to great lengths to hide their reactions. In either case, explaining the ultimate goal of data collection should increase acceptance.

A large body of literature describes the effects of collecting and combining additional data alongside survey responses (cf. the review by Singer 1993, as well as the recent special issue by Plutzer 2019 and the contributions therein, e.g., Keusch et al. 2019). Regarding the specific consent to augmenting responses with other data sources, one extensively investigated scenario is the linkage of survey data to individuals’ administrative records. Here, too, researchers have extensively investigated which factors influence respondents’ willingness to agree to data linkage. For example, highlighting the benefits to be gained (Sakshaug and Kreuter 2013, 2014), or losses to be avoided (Kreuter, Sakshaug, and Tourangeau 2016), increases consent rates, though this may depend on other survey features such as the point at which consent is elicited (Sakshaug, Tutz, and Kreuter 2013; Fobia et al. 2019; Sakshaug et al. 2019).

For mouse-tracking data more specifically, the existing literature on attitudes toward paradata usage in online surveys may provide starting points for interventions. In particular, Couper and Singer (2013) investigated the effects of paradata collection on participants’ reported willingness to participate in a survey (using keystrokes and time stamps as examples). They found that collecting paradata reduced willingness to participate, an effect that was only partially (and across studies not consistently) offset by providing a motivation or justification for the additional data collection. Regarding the willingness to consent to paradata use, specific motivations or explanations proved to be somewhat advantageous in one of the studies (compared to providing no reasons), but not in the other. In a similar vein, Kunz and Gummer (2019) examined attitudes toward paradata use in web surveys concerning three types of data: the type of device used, time stamps for mouse clicks, and geolocation information. Their results varied considerably across the different types of data, underlining the importance of investigating further paradata sources individually.

So far, research on participants’ privacy attitudes toward recording their interactions within an online survey have not considered mouse tracking despite its unique privacy implications. Therefore, we investigate willingness to participate in a study that collects participants’ cursor movements, and how best to frame and explain data collection in light of benign (scientific) goals.

In our study, we extend experiments II and III by Couper and Singer (2013). As in the original paper, we observe prospective respondents’ willingness to participate in a hypothetical survey, with or without paradata collection, and vary the stated reasons of different specificity for the measure (adopted from the original article). Unlike Couper and Singer (2013), we focus specifically on mouse movement data (which were not investigated in the original article). We also distinguish between the joint assessment of willingness to participate for both the survey in general and mouse tracking (on the one hand), and a separate consent to mouse tracking following the overall survey consent (on the other).

## Methods and Hypotheses

### Data

Our experiment was fielded by nonprobability panel provider Respondi AG, who recruited and remunerated 1,504 respondents as part of a larger online study on privacy attitudes and data use more generally in July 2019. The items and manipulations reported herein were presented as a self-contained block following the larger experiment (see Gerdon et al. 2021, for a detailed discussion of the sampling process and preceding study, and Supplementary Material section S2 for a full listing of AAPOR disclosure elements). Our assignment to conditions was orthogonal to manipulations in the remainder of the study to preclude confounding.

The sample was drawn to represent the German population with regard to age and gender through independent quotas. Of the responses, we excluded three repeat participants and three breakoffs. The final sample consisted of 1,498 members of the German public (750 female, 748 male, between 18 and 69 years of age, *M *=* *44.3 years, *SD *=* *14.3) and, given our focus on the experimental manipulation rather than a population parameter, was not weighted further.

### Design

**Figure 1.**
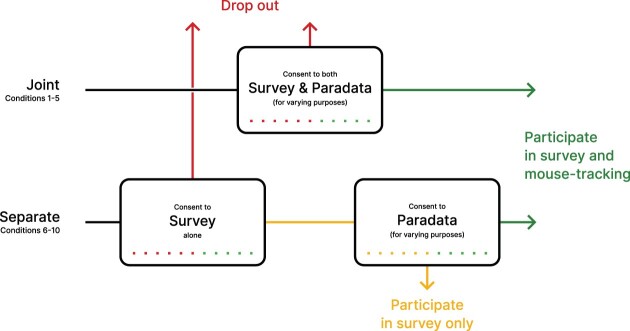
Conceptual overview of the study design. Participants were assigned to either the joint or separate consent conditions (top and bottom row), where they assessed one or two vignettes, respectively. Respondents reported their willingness to participate on an 11-point scale, which we analyze as a numeric value. Because, in a survey, consent is a binary choice, we dichotomize the ratings above the scale midpoint and assume that participants would progress if they responded above this value. Otherwise, we assumed they would drop out of the study entirely (red) or consent to the survey but not paradata collection (yellow).

In order to assess participants’ reported willingness to participate (RWTP) under different scenarios, we conducted a vignette experiment that manipulated the structure of the consent elicitation process, and the motivation provided for collecting mouse-tracking data (figure 1). In all conditions, respondents were asked to imagine a hypothetical online questionnaire covering social and political questions, conducted by a university research institute. They had been invited to participate by email and would be reimbursed with five euros for around 15 minutes of their time. For all participants, the consent was split across two screens, the first of which assessed participants’ willingness to take part in the study overall, followed by a screen that asked for consent to mouse tracking specifically. Both measured self-reported willingness to participate on an 11-point scale (from 0 to 10, with higher values indicating more positive attitudes).

The experimental manipulations (table 1) concerned, first, whether mouse tracking was introduced jointly as part of the general consent (within the same text, conditions 1–5), or separately as a distinct choice independent of the decision to complete the study (on a second, distinct, page, conditions 6–10). Comparing the results allows us to evaluate whether mere mention of mouse-tracking data collection reduces RWTP compared to a vignette limited to responses only.^2^

**Table 1.** Overview of conditions in the survey.

Consent elicitation	Purpose for mouse-tracking data collection	Condition
Joint consent/willingness to participate for survey and mouse-tracking data collection	None	1
	Scientific research purposes	2
	Understanding responses	3
	Improving survey	4
	Understanding responses and improving survey	5

Separate/independent consent to survey and mouse-tracking data collection	None	6
	Scientific research purposes	7
	Understanding responses	8
	Improving survey	9
	Understanding responses and improving survey	10

*Note:* The two experimental factors concern (first) the position at which the recording of mouse movements is mentioned, either jointly as part of the overall survey description or as an independent choice following the general willingness to participate in the survey, and (second) the purpose given for collecting mouse movements.

A second, orthogonal experimental factor varied the motivation provided for collecting mouse movement data, adapted from Couper and Singer (2013). In roughly increasing order of specificity, mouse tracking was introduced without an explanation (conditions 1 and 6), with a generic reference to scientific research purposes not further specified (conditions 2 and 7), or with the more explicit motivations of better understanding the responses, improving the survey, or both (conditions 3–5 and 8–10).

### Hypotheses

**Figure 2.**
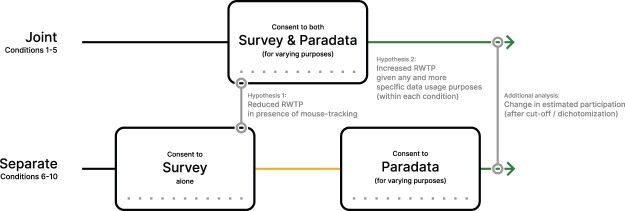
Conceptual overview of our hypotheses and analyses. We compare reported willingness to participate between a survey-only consent and a joint consent in which paradata collection is included, assuming that paradata collection would reduce willingness to participate (H1). We then compare different reasons given for paradata collection, assuming that the presence of a reason, and additional specificity, will increase willingness to participate (H2). Finally, we estimate overall participation rates in the survey alone or in combination with paradata collection, by dichotomizing our continuous measure of willingness to participate.

In line with previous findings that adding subjective “costs” decreases participation (e.g., Singer 2011), we hypothesize (H1, figure 2) that including mouse movement collection in the study description (joint consent, conditions 1–5) will reduce RWTP, compared to a study that does not include mouse-tracking data collection as part of its initial consent (conditions 6–10). Following the notion of Contextual Integrity, we expect that explaining the goals of mouse-tracking data collection increases RWTP (H2). Doing so creates an appropriate flow of information, in that data collection and use occur within a related context in service of meaningful purposes and values of the domain in which the exchange takes place (Gerdon et al. 2021). Specifically, we expect that any explanation increases RWTP over none (conditions 1 < 2–5 and 6 < 7–10), and more concrete goals fare better than vague statements (conditions 2 < 3–5 and 7 < 8–10).

### Analysis

To ascertain the effect of consent structure on RWTP and consent to paradata collection, we computed either linear regression models with the numeric response as dependent variable, or—because consent is ultimately binary—logistic models with a dichotomized dependent variable, split mid-scale^3^ (treating values greater than 5 as consent, as in Kunz and Gummer 2019). Dichotomizing respondents’ continuous responses also allows us to combine multiple items in the split consent condition: in particular, we approximate the proportion of participants willing to consent to both the survey as a whole *and* paradata collection by computing the proportion of participants whose responses on both items lay above the threshold. As we discuss below, this is a rough and imperfect estimate.

For either analysis variant, the contrast-coded predictors (based on adapted Helmert contrasts, cf. Schad et al. 2020), represented the experimental condition, comparing the joint versus independent consent conditions, and the stated reasons for paradata collection within these conditions.^4^

## Results

Table 2 shows the reported willingness to participate and consent for the three items we assessed between our two experimental groups, two for the separate and one for the joint consent condition.

**Table 2.** Descriptive results for reported willingness to participate (RWTP) across the three main questions in our experiment, by content and condition.

RWTP in …	Condition	n	Mean	SD	Q_1_	Mdn	Q_3_
Survey and mouse tracking (joint)	Joint, 1–5	744	7.22	3.17	5	8	10
Survey only (no mention of mouse tracking)	Separate, 6–10	754	8.36	2.32	8	10	10
Mouse tracking after survey (separately)	Separate, 6–10	754	6.55	3.36	5	7	10

**Figure 3.**
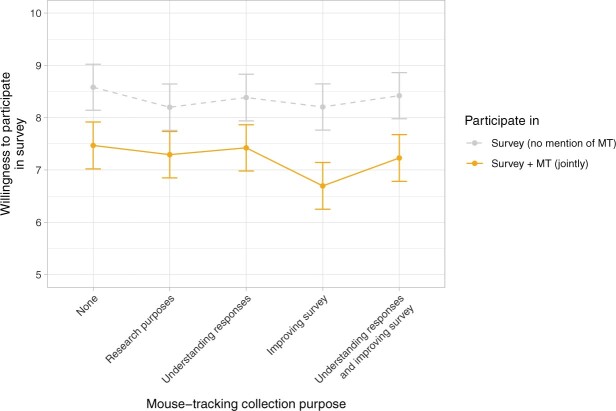
Mean willingness to participate in the survey (as a continuous scale, 0–10) separately for participants who were informed about mouse-tracking data (conditions 1–5, solid orange line) collection or not (conditions 6–10, dashed grey line), depending on the paradata collection purpose. Error bars indicate 95% confidence intervals. Please note that the conditions 6–10 had not yet been exposed to the different stated purposes for mouse tracking; they may serve as an indicator of the variability between groups under identical conditions.

Our first hypothesis concerned the effect of including mouse tracking in the overall study consent, that is, whether mouse tracking was part of the general study description and therefore mandatory, as opposed to a study description without paradata. RWTP (figure 3) was significantly reduced by including mouse tracking in the study vignette (conditions 1–5) compared to a vignette that made no mention of mouse tracking (conditions 6–10), *b *=* *1.14, 95% CI = [0.85, 1.42], *t*(1496) = 7.94, *p* < .001. This result also holds if RWTP is dichotomized: more potential respondents were willing to participate when the study vignette did not include mouse tracking (86 percent) than when it did (75 percent), log odds change = 0.73, 95% CI = [0.46, 1.00], *z *=* *5.41, *p* < .001.^5^

**Figure 4.**
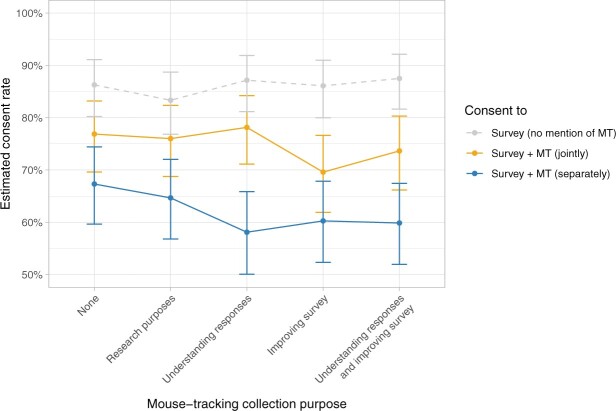
Overall willingness to participate in the survey, in proportion of participants indicating a willingness to participate above the scale midpoint. The different lines compare the willingness to participate in the survey in general, drawn from the separate consent condition, where mouse tracking was not included in the initial consent (light gray dashed line, as in figure 3) and consent rates to mouse-tracking data collection specifically (solid lines) for joint (orange) and separate (blue) conditions split by paradata collection purpose (X-axis). Error bars indicate 95 percent confidence intervals.

To approximate the effective proportion of participants who would provide mouse-tracking data in the independent consent condition, we limit ourselves to the 86 percent of this group who were willing to participate in the study at all. For these, the willingness to also provide mouse-tracking data was 7.03 (*SD *=* *3.18) on average, with 72 percent of participants indicating assent above the scale midpoint. As a result, the overall share of participants in this condition willing both to take part in the study and consent to mouse-tracking data collection was 62 percent. This overall consent rate (figure 4) was significantly lower than in the condition that jointly assessed RWTP in the survey with mouse tracking (75 percent), log odds change = –0.60, 95% CI = [–0.83,–0.37], *z *=* *–5.30, *p* < .001.

To investigate the effect of the various purposes for mouse-tracking data collection (H2), we repeated the previous analyses separately for the different consent elicitation conditions, including the purpose for mouse-tracking data collection as predictor.

For the joint consent condition, not specifying any reason for recording mouse movements unexpectedly led to the highest reported RWTP descriptively, as can be seen in figure 3. When contrasting the different purposes in the linear model, however none of the expected differences were detectable (results reported in table 3). Notably, the purpose of improving the survey resulted in a significantly *lower* RWTP compared to the purpose of better understanding responses (conditions 3 vs. 4). In a logistic regression predicting an RWTP greater than 5, none of the purpose contrasts resulted in a significant effect.

**Table 3.** Results when predicting the reported willingness to participate in the joint consent condition contrasting the different purpose manipulations in a linear or logistic regression (willingness > 5).

Linear model					
	Contrast	*b*	95% CI	*t*	*p*
No reason vs. any	(1 vs. 2–5)	−0.31	[−0.88, 0.26]	−1.06	.289
Vague vs. specific reasons	(2 vs. 3–5)	−0.18	[−0.76, 0.41]	−0.59	.554
One vs. both specific reasons	(3–4 vs. 5)	0.17	[−0.45, 0.79]	0.53	.593
Understanding responses vs. improving surveys	(3 vs. 4)	−0.73	[−1.45, −0.01]	−1.99	.047

Logistic regression model					
	Contrast	log odds Δ	95% CI	*z*	*p*
No reason vs. any	(1 vs. 2–5)	−0.13	[−0.57, 0.29]	−0.60	.548
Vague vs. specific reasons	(2 vs. 3–5)	−0.11	[−0.55, 0.31]	−0.50	.619
One vs. both specific reasons	(3–4 vs. 5)	−0.02	[−0.47, 0.43]	−0.10	.919
Understanding responses vs. improving surveys	(3 vs. 4)	−0.45	[−0.97, 0.07]	−1.68	.093

*Note:* The models contrast the different purpose manipulations in a linear and a logistic regression (predicting willingness to consent > 5). Condition numbers for the contrast variables correspond to those in table 1, and contrasts are coded such that positive coefficients are in line with the hypotheses: the reason listed first was given a negative, and the second a positive contrast in the model.

For the independent consent condition, we limited the analysis to participants who had previously indicated willingness to participate in the survey as a whole. For these, providing any reason for mouse tracking led to a significantly lower willingness to consent to paradata collection than when no reason was provided (as shown in table 4), again contrary to our initial hypotheses. None of the other contrasts had a significant effect. In a logistic regression predicting a level of willingness to consent above mid-scale, only adding a specific reason had a statistically significant effect, but again *reducing* consent rates.^6^

**Table 4.** Willingness to consent to mouse tracking in the independent consent condition, given prior willingness to participate in the survey as a whole (limiting the analysis to participants with willingness to participate in the survey > 5).

Linear model					
	Contrast	*b*	95% CI	*t*	*p*
No reason vs. any	(6 vs. 7–10)	−0.65	[−1.26, −0.04]	−2.10	.037
Vague vs. specific reasons	(7 vs. 8–10)	−0.56	[−1.20, 0.08]	−1.73	.083
One vs. both specific reasons	(8–9 vs. 10)	0.34	[−0.32, 1.01]	1.02	.310
Understanding responses vs. improving surveys	(8 vs. 9)	−0.07	[−0.84, 0.71]	−0.17	.866

Logistic regression model					
	Contrast	log odds Δ	95% CI	*z*	*p*
No reason vs. any	(6 vs. 7–10)	−0.38	[−0.85, 0.06]	−1.63	.103
Vague vs. specific reasons	(7 vs. 8–10)	−0.47	[−0.96, −0.01]	−1.96	.050
One vs. both specific reasons	(8–9 vs. 10)	0.00	[−0.44, 0.46]	0.01	.990
Understanding responses vs. improving surveys	(8 vs. 9)	0.15	[−0.37, 0.68]	0.58	.564

*Note:* Condition numbers for the contrast variables correspond to those in table 1, and contrasts are coded such that positive coefficients are in line with the hypotheses: the reason listed first was given a negative, and the second a positive contrast in the model.

Finally, to investigate whether different participants might respond differently based on their demographic characteristics, we repeated the previous analyses, including the available demographic data, namely participants’ gender and age, as additional predictors. We found no significant main effects, nor an interaction with the manipulations for any of the demographic variables.^7^

## Discussion

Capturing the interaction of participants with an online survey through mouse movements promises to improve data quality. However, it may require respondents’ explicit and specific consent. Our aim herein was to investigate the extent of this consent among panel participants, and how best to communicate the request for collecting mouse-tracking data.

In a vignette experiment, we found that participants reported generally lower willingness to take part in a survey that included mouse-tracking paradata collection. However, making mouse tracking a mandatory part of the overall consent resulted in a higher estimated proportion of participants consenting to mouse tracking compared to a separate opt-in.

The implication from our results is that the consent structure should be chosen to match survey aims: if the goal is to maximize the overall number of participants, paradata collection should be an optional addition to the survey; if it is to collect the largest possible amount of paradata, a mandatory or joint consent will increase the proportion of participants providing such data.

We did not find benefits of further explaining purposes for paradata collection; if anything, they reduced willingness to participate or consent to mouse-tracking data collection. Though contrary to our hypotheses, this is broadly in line with previous results (Couper and Singer 2013; Kunz and Gummer 2019). It is possible that the finer details of the consent are lost on participants (Kreuter et al. 2018), or that participants simply did not find the generic reasons persuasive. Here, Contextual Integrity theory (Nissenbaum 2004, 2011) suggests that a more specific explanation tailored to a study’s substantive goals may increase acceptance.

This result comes with the general limitations of a vignette study: it remains to be seen whether the results translate to an actual survey setting, especially if incentives are provided for participation that respondents would forgo by not taking part. Similarly, willingness to participate, which we assessed on a continuous scale, will translate into a binary decision on the part of actual participants. In our analyses, we approximated this through additional analyses based on a dichotomized variable; however, whether our cutoff and mapping was correct is an open question. The ordering of consent rates by experimental condition remained constant across the entire range of cutoff values (see Supplementary Material). Thus, while the differences in willingness to participate as a continuous (and ordinal) variable across our manipulations indicate the effects of various forms of consent, we would advise caution in interpreting the absolute consent rates we obtained. Likewise, differences between panel members and less experienced survey participants (panel bias) or cross-cultural differences in privacy norms (e.g., Reed, Spiro, and Butts 2016; Altmann et al. 2020; Prince and Wallsten 2022), the specific explanations offered, or the data collection context may have affected results by sensitizing participants to privacy concerns. For this reason, we would again be careful about generalizations to other situations and groups especially concerning the absolute levels of consent we found—the point we want to make concerns the reduced consent following the addition of mouse-tracking paradata. We could not find main effects or interactions with the demographic variables available to us; that does, of course, not rule out that there may be relevant individual differences we did not capture. Emulating a consent process in vignette form, as we did, may have meant that the responses are not pure measures of opinion concerning mouse tracking but are affected by the elicitation format. As our reviewers rightly pointed out, it is possible that a two-step consent process exacerbates the difference in consent due to the pragmatic interpretation of the two questions: asking for the RWTP for the survey as a whole followed by the RWTP for mouse tracking specifically in direct succession may have led participants to differentiate their responses more than they would have otherwise, to the degree that participants perceived the questions as overlapping (Strack, Schwarz, and Wänke 1991). Conversely, in the single-page consent, mouse tracking may not have influenced participants’ RWTP as strongly given that it was only one piece of information out of many regarding the survey (a dilution effect; see Nisbett, Zukier, and Lemley 1981; Tetlock, Lerner, and Boettger 1996). We would argue that these effects are likely to apply to real-world consent to the same extent, though this is clearly an empirical question.

Going forward, another solution for privacy issues surrounding paradata may be to not save the data in the first place. For example, future paradata collection tools may extract and save only specific features of interest, reducing privacy concerns. As a further step, real-time interventions that prompt participants to revisit their responses based on paradata may make storing the mouse-tracking data (and consent to this storage) unnecessary; the data may be deleted immediately after the survey, or not leave participants’ devices at all (e.g., Konečný et al. 2016).

The field of survey paradata remains in flux, and mouse tracking particularly so. It is not entirely surprising that some participants are skeptical of an unknown technique. Nevertheless, the majority of participants across all conditions in our study were open to the collection of mouse-tracking data, and we expect that proportion to grow as mouse tracking becomes more common.

## Supplementary Material

nfad034_Supplementary_Data

## Data Availability

Replication data as well as the full analysis code and results are available on the Open Science Framework at https://osf.io/kvrxa.

## Footnotes

1 For example, in Germany alone, the gesis research institute classifies interaction data as nonpersonal paradata, and notes that “explicit consent for collecting and using nonpersonal web paradata is not required” (Kunz et al. 2020, p. 4), and recommends informing participants of the fact that the data is collected. At the same time, the German Society for Online Research requires “explicit and active consent” (German Society for Online Research 2021, p. 2), particularly if paradata are linked to responses, though they note that informing participants may be sufficient if the data are used purely for survey management or quality control.

2 We disregard, for the purposes of this paper, the second page added in the joint consent condition to balance the questionnaire length. It concerned the (double hypothetical) willingness to participate in paradata collection, had participants been given the independent choice. Data for this question are available as part of the study’s replication data.

3 In the Supplementary Material, we perform sensitivity analyses and show that our pattern of results is independent of the specific split. We also report ordinal models that better take into account the ordinal nature of the Likert scale responses and avoid the loss of data associated with dichotomizing responses. We additionally investigate interactions with individual characteristics.

4 We make all of our data (in anonymized form), analysis code, and results available at https://osf.io/kvrxa, and also report extensive supplementary analyses therein. In particular, the full models as well as our contrast coding scheme are presented in detail.

5 The effect also replicates if RWTP is treated as an ordinal instead of a linear criterion; see Supplementary Material.

6 As in the linear model, no effect emerged when analyzing all participants in the separate consent condition, and when predicting the willingness to both participate in the survey and consent to mouse-tracking data collection (both > 5). Ordinal models, likewise, did not detect effects of the purpose stated in the study vignette on RWTP.

7 The specific analyses and detailed results are available in the Supplementary Material.
