# Supplementary material for: Privacy Attitudes toward Mouse-Tracking Paradata Collection
Source: Public Opin Q. 2023 Aug 9;87(Suppl 1):602–18. doi: 10.1093/poq/nfad034 (PMC10496572; doi:10.1093/poq/nfad034)
Supplement: nfad034_Supplementary_Data [file nfad034_supplementary_data.pdf]

Supplementary Material

Privacy attitutes toward mouse-tracking paradata collection

Felix Henninger, Pascal Kieslich,
Amanda Fernández-Fontelo,
Sonja Greven and Frauke Kreuter

S 1 Instructions provided to respondents

The following instructions are translated from the German questionnaire:

S 1.1 Conditions 1-5, page 1

Please imagine that you received the following email invitation to an online survey by a university research facility:

"We would like to ask you to participate in an online survey, which will capture your opinions around social and political questions. Your answers are confidential. The survey will take around 15 minutes and you will receive 5 € for your participation.

In addition to your answers, we will record your mouse movements as you complete the survey [REASON, see below]. As soon as you have completed the survey, we will stop doing so. We will never collect your mouse movements outside of the survey. Like your answers, the collected mouse movements are treated confidentially."

On a scale from 0 to 10, where 0 represents "would definitively not take part" and 10 "would definitely take part", to what degree would you be willing to participate in the survey? [RATING SCALE from 0 to 10]

S 1.2 Conditions 1-5, page 2

In future surveys of this nature, you may be asked separately whether you consent to the recording of your mouse movements [REASON, see below].

On a scale from 0 to 10, where 0 represents "would definitively not agree" and 10 "would definitely agree", to what degree would you be willing to agree to the recording of your mouse movements during the survey? [RATING SCALE from 0 to 10]

S 1.3 Conditions 6-10, Page 1

Please imagine that you received the following email invitation to an online survey by a university research facility:

"We would like to ask you to participate in an online survey, which will capture your opinions around social and political questions. Your answers are confidential. The survey will take around 15 minutes and you will receive 5 € for your participation."

On a scale from 0 to 10, where 0 represents "would definitively not take part" and 10 "would definitely take part", to what degree would you be willing to participate in the survey? [RATING SCALE from 0 to 10]

S 1.4 Conditions 6-10, page 2

Please imagine that the invitation you received contained the following additional information:

"In addition to your answers, we will record your mouse movements as you complete the survey [REASON, see below]. As soon as you have completed the survey, we will stop doing so. We will never collect your mouse movements outside of the survey. Like your answers, the collected mouse movements are treated confidentially."

On a scale from 0 to 10, where 0 represents "would definitively not agree" and 10 "would definitely agree", to what degree would you be willing to agree to the recording of your mouse movements during the survey? [RATING SCALE from 0 to 10]

S 1.5 Reason varying per condition

In addition to your answers, we record your mouse movements as you complete the survey ...

- . [no further reason provided, condition 1/6]

- , to use these for scientific research. [condition 2/7]
- , to better understand your responses. [condition 3/8]
- , to improve the survey. [condition 4/9]
- , to better understand your responses and improve the survey. [condition 5/10]

S 2 AAPOR Disclosure

The study reported herein was part of a larger data collection effort, reported in detail by Gerdon et al. (2021). AAPOR disclosure items follow:

- **Data Collection Strategy**: Our data was collected as an online survey experiment.
- **Who Sponsored the Research and Who Conducted It**: Data collection was funded in part by the Volkswagen Foundation project "Consequences of Artificial Intelligence for Urban Societies", as well as the Deutsche Forschungsgemeinschaft (DFG, projects 396057129 and 139943784). Colleagues responsible for other sections of the data collection effort were also supported by DFG projects KR2211/5-1 and GR3793/2-1., the University of Mannheim's Graduate School of Economic and Social Sciences, the US National Security Agency (The Science of Privacy: Implications for Data Usage, H98230-18-D-006) and the US National Science Foundation (Contextual Integrity: From Theory to Practice, CNS-1801501).
- **Measurement Tools/Instruments**: Please see the previous section for our items, and Gerdon et al. (2021) for those of the questionnaire section preceding it.
- **Population Under Study/Method Used to Generate and Recruit the Sample**: Our sample was drawn from an opt-in, nonprobability panel managed by provider Respondi AG, with independent quotas for gender and age chosen to represent the German general population in these regards. Panel members registered to receive invitations to surveys and other data collection efforts, and received a modest recompense for their effort after completing the questionnaire.
- **Method(s) and Mode(s) of Data Collection.**: German language CAWI
- **Dates of Data Collection**: July 9th to 18th, 2019

- **Sample Sizes**: 1504 respondents, 1498 complete, unique datasets for analysis
- **How the Data Were Weighted**: No weighting was applied.
- **How the Data Were Processed and Procedures to Ensure Data Quality**: We excluded (three) repeat participants through the identifier supplied by the panel provider, and (three) participants who did not complete the experiment.
- **A General Statement Acknowledging Limitations of the Design and Data Collection**: As we note in our discussion, we focus on the experimental effect of introducing mouse-tracking, and urge caution against over-interpreting the absolute consent rates we report, which may reflect ideosyncracies of the sample or the data collection context. As with any vignette experiment, our results rest on the assumption that intentions provide a proxy for behavior, and may change in the presence of incentives for participation.

S 3 Regression models

We now report equations and summary tables for the models we discuss in the paper. In doing so, we respectfully refer our interested readers to our open analysis script for the full details of our analysis – again we provide only a summary of the results here, and kindly request that the main analysis document be regarded as canonical.

Throughout, we show standard errors in parentheses and confidence intervals in square brackets.

S 3.1 Willingness to participate in study overall

As in the paper, we first look at the reported willingness to participate in the study overall (assessed on page one), split by joint (the consent includes both the survey overall and the mouse-tracking specifically on this first page) vs independent (mouse-tracking consent is given on the second page) consent condition. At this point, we collapse across the second experimental factor, the stated reasons for including mouse-tracking in the survey.

S 3.1.1 Linear model / Continuous

The first model we report concerns the effect of the presence of mouse-tracking in the initial consent ($consent_1$), on the reported willingness to participate as a continuous variable (*response*_1):

$$\text{response\_1} = \alpha + \beta_1(\text{consent}_1) + \epsilon \quad (1)$$

The output table for this result is as follows:

Table 1:

	(1)
(Intercept)	7.79*** (0.07)
	[7.65, 7.93]
consent1	1.14*** (0.14)
	[0.86, 1.42]
Num.Obs.	1498
R2	0.040
R2 Adj.	0.040
AIC	7307.9
BIC	7323.8
Log.Lik.	−3650.927
RMSE	2.77

s 3.1.2 Logistic model / Dichotomized

We can also dichotomize the scale above its center point, resulting in a logistic model:

$$\log\left[\frac{P(\text{response\_1} > 5)}{1 - P(\text{response\_1} > 5)}\right] = \alpha + \beta_1(\text{consent}_1)$$

This yields the following results:

Table 2:

	(1)
(Intercept)	1.46*** (0.07)
	[1.33, 1.59]
consent1	0.73*** (0.13)
	[0.47, 1.00]
Num.Obs.	1498
AIC	1451.6
BIC	1462.2
RMSE	0.39

s 3.1.3 Including demographic variables

As an additional exploratory analysis, we included the available demographic characteristics of every participant, age and gender, and investigate their main effects and pairwise interactions with the first experimental factor

S 3.1.3.1 Linear

$$
\begin{aligned}
\text{response\_1} = \alpha + \\
& \beta_1(\text{consent}_1) + \\
& \beta_2(\text{age\_c}) + \\
& \beta_3(\text{sex}_{\text{male\_vs\_female}}) + \\
& \beta_4(\text{consent}_1 \times \text{age\_c}) + \\
& \beta_5(\text{consent}_1 \times \text{sex}_{\text{male\_vs\_female}}) + \\
& \epsilon
\end{aligned}
\tag{2}
$$

Table 3:

	(1)
(Intercept)	7.79*** (0.07)
	[7.65, 7.93]
consent1	1.13*** (0.14)
	[0.85, 1.41]
age_c	0.00 (0.01)
	[−0.01, 0.01]
sexmale_vs_female	0.21 (0.14)
	[−0.07, 0.50]
consent1 × age_c	0.01 (0.01)
	[−0.01, 0.03]
consent1 × sexmale_vs_female	−0.37 (0.29)
	[−0.93, 0.20]
Num.Obs.	1498
R2	0.044
R2 Adj.	0.041
AIC	7310.2
BIC	7347.3
Log.Lik.	−3648.076
RMSE	2.76

s 3.1.3.2 Logistic

$$\log\left[\frac{P(\text{response\_1} > 5)}{1 - P(\text{response\_1} > 5)}\right] = \alpha +$$

$$\beta_1(\text{consent}_1) +$$

$$\beta_2(\text{age\_c}) +$$

$$\beta_3(\text{sex}_{\text{male\_vs\_female}}) +$$

$$\beta_4(\text{consent}_1 \times \text{age\_c}) +$$

$$\beta_5(\text{consent}_1 \times \text{sex}_{\text{male\_vs\_female}})$$

Table 4:

	(1)
(Intercept)	1.46*** (0.07)
	[1.33, 1.60]
consent1	0.72*** (0.14)
	[0.46, 0.99]
age_c	0.00 (0.00)
	[−0.01, 0.01]
sexmale_vs_female	0.19 (0.14)
	[−0.08, 0.45]
consent1 × age_c	0.01 (0.01)
	[−0.01, 0.03]
consent1 × sexmale_vs_female	−0.12 (0.27)
	[−0.66, 0.41]
Num.Obs.	1498
AIC	1454.4
BIC	1486.3
RMSE	0.39

s 3.2 Willingness to participate in survey *and* mouse-tracking

We now focus on the proportion of participants who would provide mouse-tracking data (*consent_q_mt*). We do so by investigating the proportion of participants who agree to both in one step, or agree to both participating in the survey *and* subsequently to mouse-tracking data collection.

$$\log\left[\frac{P(\text{consent\_q\_mt} = \text{TRUE})}{1 - P(\text{consent\_q\_mt} = \text{TRUE})}\right] = \alpha + \beta_1(\text{consent}_1) \tag{3}$$

Table 5:

	(1)
(Intercept)	0.79*** (0.06)
	[0.68, 0.90]
consent1	−0.60*** (0.11)
	[−0.82, −0.38]
Num.Obs.	1498
AIC	1843.8
BIC	1854.5
Log.Lik.	−919.921
RMSE	0.46

Again, we can expand these models taking into account demographic information as covariates:

$$\log\left[\frac{P(\text{consent\_q\_mt} = \text{TRUE})}{1 - P(\text{consent\_q\_mt} = \text{TRUE})}\right] = \alpha + \beta_1(\text{consent}_1) + \beta_2(\text{age\_c}) + \beta_3(\text{sex}_{\text{male\_vs\_female}}) + \beta_4(\text{consent}_1 \times \text{age\_c}) + \beta_5(\text{consent}_1 \times \text{sex}_{\text{male\_vs\_female}}) \tag{4}$$

Table 6:

	(1)
(Intercept)	0.80*** (0.06)
	[0.69, 0.91]
consent1	−0.61*** (0.11)
	[−0.83, −0.39]
age_c	0.00 (0.00)
	[−0.01, 0.01]
sexmale_vs_female	0.20 (0.11)
	[−0.02, 0.42]
consent1 × age_c	0.01 (0.01)
	[0.00, 0.03]
consent1 × sexmale_vs_female	−0.10 (0.23)
	[−0.54, 0.35]
Num.Obs.	1498
AIC	1845.4
BIC	1877.2
Log.Lik.	−916.681
RMSE	0.46

S 3.3 Effect of provided reasons

In addition to the structure of the consent elicitation process, we also vary the stated reasons provided for conducting mouse-tracking paradata collection. As we explain in the paper, we evaluated multiple potential explanations adapted from Cooper & Singer (2013) that range from vague and generic to more specific and concrete. We expected, but did not find, a positive influence of additional motivation and reasoning around paradata collection.

We split the analysis here by our first experimental factor, because the data were collected on two different survey pages, and either include willingness to participate in the survey overall (joint consent) or concern willingness to participate in mouse-tracking independent of survey participation (separate consent condition). For the separate consent condition, we further restrict the sample to participants who indicated willingness to complete the survey overall.

S 3.3.1 Joint consent condition

These models are summarized in the paper in Table 3.

S 3.3.1.1 Linear model

$$
\begin{aligned}
\text{response\_1} = \alpha + \\
&\beta_1(\text{purpose}_{\text{No vs. any reason}}) + \\
&\beta_2(\text{purpose}_{\text{Generic vs. specific}}) + \\
&\beta_3(\text{purpose}_{\text{One vs. both}}) + \\
&\beta_4(\text{purpose}_{\text{Understand vs. improve}}) + \\
&\epsilon
\end{aligned}
\tag{5}
$$

Table 7:

	(1)
(Intercept)	7.22*** (0.12)
	[6.99, 7.45]
purposeNo vs. any reason	−0.31 (0.29)
	[−0.88, 0.26]
purposeGeneric vs. specific	−0.18 (0.30)
	[−0.76, 0.41]
purposeOne vs. both	0.17 (0.32)
	[−0.45, 0.79]
purposeUnderstand vs. improve	−0.73* (0.37)
	[−1.45, −0.01]
Num.Obs.	744
R2	0.008
R2 Adj.	0.002
AIC	3831.3
BIC	3859.0
Log.Lik.	−1909.647
RMSE	3.15

Taking into account demographic variables once more:

$$\begin{aligned}
\text{response_1} = \alpha + \\
&\beta_1(\text{purpose}_{\text{No vs. any reason}}) + \\
&\beta_2(\text{purpose}_{\text{Generic vs. specific}}) + \\
&\beta_3(\text{purpose}_{\text{One vs. both}}) + \\
&\beta_4(\text{purpose}_{\text{Understand vs. improve}}) + \\
&\beta_5(\text{age_c}) + \\
&\beta_6(\text{sex}_{\text{male_vs_female}}) + \\
&\beta_7(\text{purpose}_{\text{No vs. any reason}} \times \text{age_c}) + \\
&\beta_8(\text{purpose}_{\text{Generic vs. specific}} \times \text{age_c}) + \\
&\beta_9(\text{purpose}_{\text{One vs. both}} \times \text{age_c}) + \\
&\beta_{10}(\text{purpose}_{\text{Understand vs. improve}} \times \text{age_c}) + \\
&\beta_{11}(\text{purpose}_{\text{No vs. any reason}} \times \text{sex}_{\text{male_vs_female}}) + \\
&\beta_{12}(\text{purpose}_{\text{Generic vs. specific}} \times \text{sex}_{\text{male_vs_female}}) + \\
&\beta_{13}(\text{purpose}_{\text{One vs. both}} \times \text{sex}_{\text{male_vs_female}}) + \\
&\beta_{14}(\text{purpose}_{\text{Understand vs. improve}} \times \text{sex}_{\text{male_vs_female}}) + \\
&\epsilon
\end{aligned} \tag{6}$$

Table 8:

	(1)
(Intercept)	7.22*** (0.12)
	[6.99, 7.45]
purposeNo vs. any reason	−0.33 (0.30)
	[−0.91, 0.25]
purposeGeneric vs. specific	−0.19 (0.30)
	[−0.78, 0.40]
purposeOne vs. both	0.19 (0.32)
	[−0.44, 0.81]
purposeUnderstand vs. improve	−0.78* (0.37)
	[−1.50, −0.06]
age_c	−0.01 (0.01)
	[−0.02, 0.01]
sexmale_vs_female	0.43 (0.23)
	[−0.03, 0.89]
purposeNo vs. any reason × age_c	0.00 (0.02)
	[−0.04, 0.03]
purposeGeneric vs. specific × age_c	0.00 (0.02)
	[−0.04, 0.04]
purposeOne vs. both × age_c	0.00 (0.02)
	[−0.04, 0.04]
purposeUnderstand vs. improve × age_c	0.00 (0.03)
	[−0.05, 0.05]
purposeNo vs. any reason × sexmale_vs_female	0.29 (0.59)
	[−0.87, 1.45]
purposeGeneric vs. specific × sexmale_vs_female	0.20 (0.60)
	[−0.98, 1.37]
purposeOne vs. both × sexmale_vs_female	−0.89 (0.64)
	[−2.15, 0.36]
purposeUnderstand vs. improve × sexmale_vs_female	0.19 (0.74)
	[−1.26, 1.63]
Num.Obs.	744
R2	0.017
R2 Adj.	−0.002
AIC	3844.2
BIC	3918.0
Log.Lik.	−1906.093
RMSE	3.14

S 3.3.1.2 Logistic model

$$\log\left[\frac{P(\text{response\_1} > 5)}{1 - P(\text{response\_1} > 5)}\right] = \alpha +$$

$$\beta_1(\text{purpose}_{\text{No vs. any reason}}) +$$

$$\beta_2(\text{purpose}_{\text{Generic vs. specific}}) +$$

$$\beta_3(\text{purpose}_{\text{One vs. both}}) +$$

$$\beta_4(\text{purpose}_{\text{Understand vs. improve}})$$

Table 9:

	(1)
(Intercept)	1.10*** (0.08)
	[0.93, 1.27]
purposeNo vs. any reason	−0.13 (0.22)
	[−0.57, 0.29]
purposeGeneric vs. specific	−0.11 (0.22)
	[−0.55, 0.31]
purposeOne vs. both	−0.02 (0.23)
	[−0.47, 0.43]
purposeUnderstand vs. improve	−0.45 (0.27)
	[−0.97, 0.07]
Num.Obs.	744
AIC	845.4
BIC	868.5
RMSE	0.43

Accounting for available demographic variables:

$$
\begin{aligned}
\log\left[\frac{P(\text{response\_1} = 9)}{1 - P(\text{response\_1} = 9)}\right] = \alpha +& \\
& \beta_1(\text{purpose}_{\text{No vs. any reason}}) + \\
& \beta_2(\text{purpose}_{\text{Generic vs. specific}}) + \\
& \beta_3(\text{purpose}_{\text{One vs. both}}) + \\
& \beta_4(\text{purpose}_{\text{Understand vs. improve}}) + \\
& \beta_5(\text{age\_c}) + \\
& \beta_6(\text{sex}_{\text{male\_vs\_female}}) + \\
& \beta_7(\text{purpose}_{\text{No vs. any reason}} \times \text{age\_c}) + \\
& \beta_8(\text{purpose}_{\text{Generic vs. specific}} \times \text{age\_c}) + \\
& \beta_9(\text{purpose}_{\text{One vs. both}} \times \text{age\_c}) + \\
& \beta_{10}(\text{purpose}_{\text{Understand vs. improve}} \times \text{age\_c}) + \\
& \beta_{11}(\text{purpose}_{\text{No vs. any reason}} \times \text{sex}_{\text{male\_vs\_female}}) + \\
& \beta_{12}(\text{purpose}_{\text{Generic vs. specific}} \times \text{sex}_{\text{male\_vs\_female}}) + \\
& \beta_{13}(\text{purpose}_{\text{One vs. both}} \times \text{sex}_{\text{male\_vs\_female}}) + \\
& \beta_{14}(\text{purpose}_{\text{Understand vs. improve}} \times \text{sex}_{\text{male\_vs\_female}})
\end{aligned}
\tag{7}
$$

Table 10:

	(1)
(Intercept)	1.11*** (0.09)
	[0.95, 1.29]
purposeNo vs. any reason	−0.13 (0.22)
	[−0.58, 0.29]
purposeGeneric vs. specific	−0.12 (0.22)
	[−0.57, 0.31]
purposeOne vs. both	−0.03 (0.23)
	[−0.48, 0.43]
purposeUnderstand vs. improve	−0.50 (0.27)
	[−1.04, 0.03]
age_c	−0.01 (0.01)
	[−0.02, 0.00]
sexmale_vs_female	0.27 (0.17)
	[−0.06, 0.61]
purposeNo vs. any reason × age_c	−0.01 (0.01)
	[−0.04, 0.02]
purposeGeneric vs. specific × age_c	0.00 (0.02)
	[−0.03, 0.03]
purposeOne vs. both × age_c	0.00 (0.02)
	[−0.04, 0.03]
purposeUnderstand vs. improve × age_c	0.01 (0.02)
	[−0.03, 0.04]
purposeNo vs. any reason × sexmale_vs_female	0.07 (0.44)
	[−0.81, 0.94]
purposeGeneric vs. specific × sexmale_vs_female	0.08 (0.44)
	[−0.80, 0.94]
purposeOne vs. both × sexmale_vs_female	−0.37 (0.46)
	[−1.27, 0.54]
purposeUnderstand vs. improve × sexmale_vs_female	−0.27 (0.54)
	[−1.34, 0.79]
Num.Obs.	744
AIC	858.8
BIC	928.0
RMSE	0.43

S 3.3.2 Independent/separate consent condition

These models are summarized in the paper in Table 4. They are limited to participants who were more willing to participate in the study overall in the first step ($WTP > 5$).

S 3.3.2.1 Linear model

$$\begin{aligned} \text{response\_2} = \alpha\ + & \\ & \beta_1(\text{purpose}_{\text{No vs. any reason}}) + \\ & \beta_2(\text{purpose}_{\text{Generic vs. specific}}) + \\ & \beta_3(\text{purpose}_{\text{One vs. both}}) + \\ & \beta_4(\text{purpose}_{\text{Understand vs. improve}}) + \\ & \epsilon \end{aligned} \tag{8}$$

Fitting the model yields the following parameters

Table 11:

	(1)
(Intercept)	7.03*** (0.12)
	[6.79, 7.28]
purposeNo vs. any reason	−0.65* (0.31)
	[−1.26, −0.04]
purposeGeneric vs. specific	−0.56 (0.33)
	[−1.20, 0.08]
purposeOne vs. both	0.34 (0.34)
	[−0.32, 1.01]
purposeUnderstand vs. improve	−0.07 (0.39)
	[−0.84, 0.71]
Num.Obs.	649
R2	0.013
R2 Adj.	0.007
AIC	3347.4
BIC	3374.2
Log.Lik.	−1667.675
RMSE	3.16

Again including demographic variables in the model:

$$
\begin{aligned}
\text{response\_2} = \alpha + \\
& \beta_1(\text{purpose}_{\text{No vs. any reason}}) + \\
& \beta_2(\text{purpose}_{\text{Generic vs. specific}}) + \\
& \beta_3(\text{purpose}_{\text{One vs. both}}) + \\
& \beta_4(\text{purpose}_{\text{Understand vs. improve}}) + \\
& \beta_5(\text{age\_c}) + \\
& \beta_6(\text{sex}_{\text{male\_vs\_female}}) + \\
& \beta_7(\text{purpose}_{\text{No vs. any reason}} \times \text{age\_c}) + \\
& \beta_8(\text{purpose}_{\text{Generic vs. specific}} \times \text{age\_c}) + \\
& \beta_9(\text{purpose}_{\text{One vs. both}} \times \text{age\_c}) + \\
& \beta_{10}(\text{purpose}_{\text{Understand vs. improve}} \times \text{age\_c}) + \\
& \beta_{11}(\text{purpose}_{\text{No vs. any reason}} \times \text{sex}_{\text{male\_vs\_female}}) + \\
& \beta_{12}(\text{purpose}_{\text{Generic vs. specific}} \times \text{sex}_{\text{male\_vs\_female}}) + \\
& \beta_{13}(\text{purpose}_{\text{One vs. both}} \times \text{sex}_{\text{male\_vs\_female}}) + \\
& \beta_{14}(\text{purpose}_{\text{Understand vs. improve}} \times \text{sex}_{\text{male\_vs\_female}}) + \\
& \epsilon
\end{aligned}
\tag{9}
$$

Table 12:

	(1)
(Intercept)	7.04*** (0.13)
	[6.79, 7.29]
purposeNo vs. any reason	−0.63* (0.31)
	[−1.25, −0.02]
purposeGeneric vs. specific	−0.60 (0.33)
	[−1.25, 0.05]
purposeOne vs. both	0.34 (0.34)
	[−0.33, 1.01]
purposeUnderstand vs. improve	−0.07 (0.40)
	[−0.85, 0.72]
age_c	0.01 (0.01)
	[−0.01, 0.02]
sexmale_vs_female	0.12 (0.25)
	[−0.38, 0.61]
purposeNo vs. any reason × age_c	0.00 (0.02)
	[−0.05, 0.04]
purposeGeneric vs. specific × age_c	−0.02 (0.02)
	[−0.07, 0.03]
purposeOne vs. both × age_c	0.00 (0.02)
	[−0.05, 0.05]
purposeUnderstand vs. improve × age_c	0.00 (0.03)
	[−0.05, 0.06]
purposeNo vs. any reason × sexmale_vs_female	0.00 (0.62)
	[−1.22, 1.23]
purposeGeneric vs. specific × sexmale_vs_female	0.02 (0.66)
	[−1.28, 1.33]
purposeOne vs. both × sexmale_vs_female	0.11 (0.69)
	[−1.25, 1.47]
purposeUnderstand vs. improve × sexmale_vs_female	0.13 (0.80)
	[−1.43, 1.69]
Num.Obs.	649
R2	0.015
R2 Adj.	−0.007
AIC	3365.9
BIC	3437.5
Log.Lik.	−1666.939
RMSE	3.16

S 3.3.2.2 Logistic model

$$\log\left[\frac{P(\text{response\_2} > 5)}{1 - P(\text{response\_2} > 5)}\right] = \alpha +$$

$$\beta_1(\text{purpose}_{\text{No vs. any reason}}) +$$

$$\beta_2(\text{purpose}_{\text{Generic vs. specific}}) +$$

$$\beta_3(\text{purpose}_{\text{One vs. both}}) +$$

$$\beta_4(\text{purpose}_{\text{Understand vs. improve}})$$

Table 13:

	(1)
(Intercept)	0.96*** (0.09)
	[0.79, 1.14]
purposeNo vs. any reason	−0.38 (0.23)
	[−0.85, 0.06]
purposeGeneric vs. specific	−0.47 (0.24)
	[−0.96, −0.01]
purposeOne vs. both	0.00 (0.23)
	[−0.44, 0.46]
purposeUnderstand vs. improve	0.15 (0.27)
	[−0.37, 0.68]
Num.Obs.	649
AIC	770.9
BIC	793.3
RMSE	0.45

Extending the model to include demographic variables:

$$
\begin{aligned}
\log\left[\frac{P(\text{response\_2} = 10)}{1 - P(\text{response\_2} = 10)}\right] = \alpha + \\
&\beta_1(\text{purpose}_{\text{No vs. any reason}}) + \\
&\beta_2(\text{purpose}_{\text{Generic vs. specific}}) + \\
&\beta_3(\text{purpose}_{\text{One vs. both}}) + \\
&\beta_4(\text{purpose}_{\text{Understand vs. improve}}) + \\
&\beta_5(\text{age\_c}) + \\
&\beta_6(\text{sex}_{\text{male\_vs\_female}}) + \\
&\beta_7(\text{purpose}_{\text{No vs. any reason}} \times \text{age\_c}) + \\
&\beta_8(\text{purpose}_{\text{Generic vs. specific}} \times \text{age\_c}) + \\
&\beta_9(\text{purpose}_{\text{One vs. both}} \times \text{age\_c}) + \\
&\beta_{10}(\text{purpose}_{\text{Understand vs. improve}} \times \text{age\_c}) + \\
&\beta_{11}(\text{purpose}_{\text{No vs. any reason}} \times \text{sex}_{\text{male\_vs\_female}}) + \\
&\beta_{12}(\text{purpose}_{\text{Generic vs. specific}} \times \text{sex}_{\text{male\_vs\_female}}) + \\
&\beta_{13}(\text{purpose}_{\text{One vs. both}} \times \text{sex}_{\text{male\_vs\_female}}) + \\
&\beta_{14}(\text{purpose}_{\text{Understand vs. improve}} \times \text{sex}_{\text{male\_vs\_female}})
\end{aligned} \tag{10}
$$

Table 14:

	(1)
(Intercept)	0.98*** (0.09)
	[0.80, 1.16]
purposeNo vs. any reason	−0.38 (0.24)
	[−0.86, 0.07]
purposeGeneric vs. specific	−0.50* (0.25)
	[−1.00, −0.03]
purposeOne vs. both	0.00 (0.23)
	[−0.45, 0.45]
purposeUnderstand vs. improve	0.16 (0.27)
	[−0.37, 0.69]
age_c	0.01 (0.01)
	[−0.01, 0.02]
sexmale_vs_female	0.15 (0.18)
	[−0.20, 0.51]
purposeNo vs. any reason × age_c	−0.01 (0.02)
	[−0.05, 0.02]
purposeGeneric vs. specific × age_c	−0.01 (0.02)
	[−0.05, 0.02]
purposeOne vs. both × age_c	0.00 (0.02)
	[−0.03, 0.03]
purposeUnderstand vs. improve × age_c	0.00 (0.02)
	[−0.04, 0.04]
purposeNo vs. any reason × sexmale_vs_female	−0.39 (0.47)
	[−1.33, 0.53]
purposeGeneric vs. specific × sexmale_vs_female	0.28 (0.49)
	[−0.68, 1.25]
purposeOne vs. both × sexmale_vs_female	0.37 (0.47)
	[−0.55, 1.29]
purposeUnderstand vs. improve × sexmale_vs_female	0.42 (0.54)
	[−0.63, 1.48]
Num.Obs.	649
AIC	786.3
BIC	853.5
RMSE	0.44

S 4 Ordinal regressions

As noted in the manuscript, we replicate our previous analysis using ordinal models, which require neither the assumption of an interval-scale response as linear models do, nor the arbitrary dichotomization that a logistic regression imposes. We omit the model equations here due to space constraints.

S 4.1 Willingness to participate in study overall

Table 15:

	(1)
0|1	-2.93^{***} (0.12)
	[−3.16, −2.70]
1|2	-2.73^{***} (0.11)
	[−2.94, −2.52]
2|3	-2.50^{***} (0.10)
	[−2.68, −2.31]
3|4	-2.32^{***} (0.09)
	[−2.50, −2.14]
4|5	-2.04^{***} (0.08)
	[−2.20, −1.88]
5|6	-1.44^{***} (0.07)
	[−1.57, −1.31]
6|7	-1.13^{***} (0.06)
	[−1.25, −1.01]
7|8	-0.72^{***} (0.06)
	[−0.82, −0.61]
8|9	-0.14^{**} (0.05)
	[−0.24, −0.04]
9|10	0.28^{***} (0.05)
	[0.17, 0.38]
consent1	0.67^{***} (0.09)
	[0.48, 0.85]
Num.Obs.	1498
AIC	5488.0
BIC	5546.4
RMSE	8.02

S 4.2 Effect of provided reasons

S 4.2.1 Joint consent condition

Fitting the model yields the following parameters

Table 16:

	(1)
0|1	−2.39*** (0.13)
	[−2.65, −2.13]
1|2	−2.23*** (0.12)
	[−2.47, −1.98]
2|3	−1.94*** (0.11)
	[−2.16, −1.72]
3|4	−1.77*** (0.10)
	[−1.97, −1.57]
4|5	−1.59*** (0.10)
	[−1.78, −1.40]
5|6	−1.09*** (0.08)
	[−1.26, −0.93]
6|7	−0.80*** (0.08)
	[−0.96, −0.64]
7|8	−0.35*** (0.07)
	[−0.49, −0.20]
8|9	0.17* (0.07)
	[0.03, 0.32]
9|10	0.55*** (0.08)
	[0.40, 0.70]
purposeNo vs. any reason	−0.14 (0.16)
	[−0.46, 0.18]
purposeGeneric vs. specific	−0.11 (0.17)
	[−0.44, 0.22]
purposeOne vs. both	0.11 (0.18)
	[−0.24, 0.46]
purposeUnderstand vs. improve	−0.39 (0.21)
	[−0.79, 0.02]
Num.Obs.	744
AIC	2985.2
BIC	3049.8
RMSE	7.67

s 4.2.2 Independent/separate consent condition

Fitting the model yields the following parameters

Table 17:

	(1)
0|1	-2.45^{***} (0.14)
1|2	-2.16^{***} (0.13)
2|3	-1.89^{***} (0.12)
3|4	-1.69^{***} (0.11)
4|5	-1.41^{***} (0.10)
5|6	-0.95^{***} (0.09)
6|7	-0.72^{***} (0.08)
7|8	-0.24^{**} (0.08)
8|9	0.32^{***} (0.08)
9|10	0.71^{***} (0.08)
purposeNo vs. any reason	−0.33 (0.17)
	[−0.67, 0.01]
purposeGeneric vs. specific	−0.29 (0.18)
	[−0.64, 0.07]
purposeOne vs. both	0.18 (0.19)
	[−0.19, 0.56]
purposeUnderstand vs. improve	0.02 (0.22)
	[−0.41, 0.45]
Num.Obs.	649
AIC	2705.6
BIC	2768.3

S 5 Robustness check for dichotomization

In the following figure, we show estimated consent rates (y axis) by threshold for dichotomization (x axis), separated by consent condition (color/line), but collapsed across stated purpose for mouse-tracking data collection. The descriptive pattern remains consistent across available threshold values:

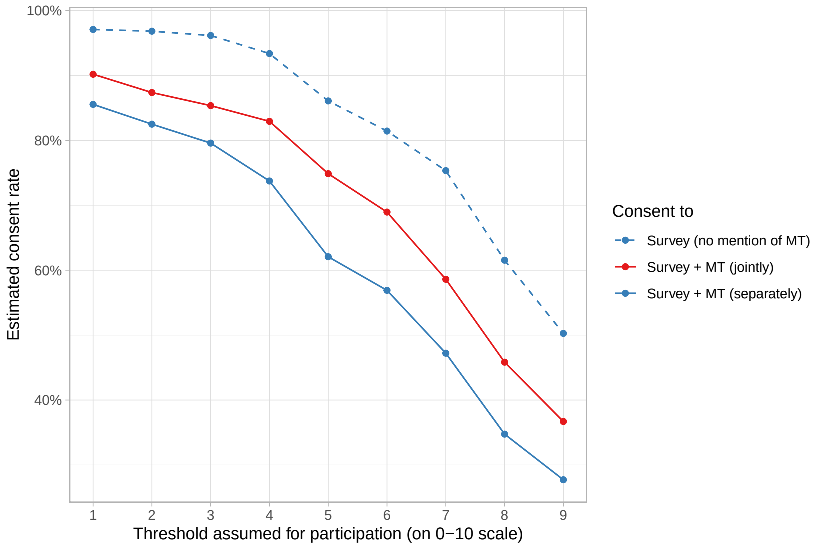

Figure 1: Responses across available choices for the dichotomization threshold
